# Evolution of Gigantism in Amphiumid Salamanders

**DOI:** 10.1371/journal.pone.0005615

**Published:** 2009-05-20

**Authors:** Ronald M. Bonett, Paul T. Chippindale, Paul E. Moler, R. Wayne Van Devender, David B. Wake

**Affiliations:** 1 Department of Biological Sciences, University of Tulsa, Tulsa, Oklahoma, United States of America; 2 Museum of Vertebrate Zoology and Department of Integrative Biology, University of California, Berkeley, California, United States of America; 3 Department of Biology, University of Texas at Arlington, Arlington, Texas, United States of America; 4 Florida Fish and Wildlife Conservation Commission, Gainesville, Florida, United States of America; 5 Department of Biology, Appalachian State University, Boone, North Carolina, United States of America; Max Planck Institute for Evolutionary Anthropology, Germany

## Abstract

The Amphiumidae contains three species of elongate, permanently aquatic salamanders with four diminutive limbs that append one, two, or three toes. Two of the species, *Amphiuma means* and *A. tridactylum*, are among the largest salamanders in the world, reaching lengths of more than one meter, whereas the third species (*A. pholeter*), extinct amphiumids, and closely related salamander families are relatively small. *Amphiuma means* and *A. tridactylum* are widespread species and live in a wide range of lowland aquatic habitats on the Coastal Plain of the southeastern United States, whereas *A. pholeter* is restricted to very specialized organic muck habitats and is syntopic with *A. means*. Here we present analyses of sequences of mitochondrial and nuclear loci from across the distribution of the three taxa to assess lineage diversity, relationships, and relative timing of divergence in amphiumid salamanders. In addition we analyze the evolution of gigantism in the clade. Our analyses indicate three lineages that have diverged since the late Miocene, that correspond to the three currently recognized species, but the two gigantic species are not each other's closest relatives. Given that the most closely related salamander families and fossil amphiumids from the Upper Cretaceous and Paleocene are relatively small, our results suggest at least two extreme changes in body size within the Amphuimidae. Gigantic body size either evolved once as the ancestral condition of modern amphiumas, with a subsequent strong size reduction in *A. pholeter*, or gigantism independently evolved twice in the modern species, *A. means* and *A. tridactylum*. These patterns are concordant with differences in habitat breadth and range size among lineages, and have implications for reproductive isolation and diversification of amphiumid salamanders.

## Introduction

Body size evolution is a key factor in generating ecological and genetic divergence, and has been a primary axis of change during the radiation of many species groups. This is because body size is a relatively labile character, yet can be important for dictating niche parameters, creating reproductive isolation, and structuring communities [1]–[5]. Furthermore, physical attributes of an organism's size can influence other ecological parameters such as dispersal capabilities and habitat specialization [6]–[9]. Some groups of organisms have experienced extreme, and often paradoxical, evolutionary changes in body-size (gigantism and miniaturization) due to colonizing new regions such as islands or deep seas [10]–[13], re-colonization after mass extinctions [14], *in situ* environmental shifts [15]–[19], or evolutionary novelty [19]. Selection for gigantic body size is favored in situations of increased resource abundance, ecological release from predators or competitors, or necessity for long distance dispersal, whereas, miniaturization often results from resource or habitat limitations.

**Figure 1:**
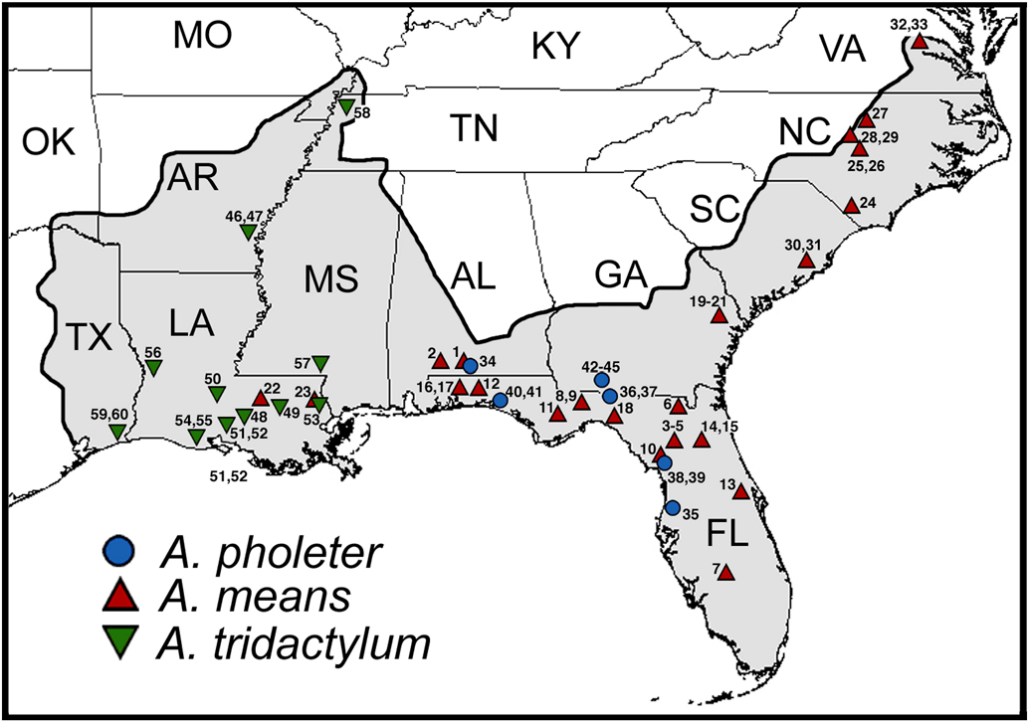
Distribution and sampling of *Amphiuma*. Map shows the combined distribution of the Amphiumidae (shaded in grey), and sampling localities for *A. pholeter* (blue circles), *A. means* (red triangles), and *A. tridactylum* (green triangles). Localities are listed in Table S1.

Salamanders of the family Amphiumidae inhabit lowland aquatic habitats throughout the Coastal Plain of the southeastern United States, and constitute an average 30 to 45 fold difference in body-size among species, including two of the largest salamanders in the world [20, this study]. Members of the genus *Amphiuma* are elongate with expanded trunks and four miniature limbs, which is a major morphological deviation from the standard salamander body plan. The three currently recognized species are diagnosed by the number of toes on each limb: the one-toed (*Amphiuma pholeter*), two-toed (*A. means*), and three-toed (*A. tridactylum*) amphiuma. *Amphiuma means* and *A. tridactylum* are truly gigantic salamanders reaching lengths of more than a meter, whereas *A. pholeter*, the smallest species, reaches only ∼36 cm in total length [20, this study]. *Amphiuma means* and *A. tridactylum* occur in a wide range of lowland aquatic habitats in the eastern and western parts of the Coastal Plain respectively (Figure 1), and may hybridize in the mid-Gulf Coastal Plain [21]. *Amphiuma pholeter* is restricted to organic muck habitats along the margin of the eastern Gulf Coastal Plain where it is microsympatric with juvenile *A. means*.

Analyzing the evolution of characteristics, such as body size, in a phylogenetic context can be fundamental to understanding the pattern and direction of change [22]. Previous molecular analyses of the Amphiumidae based on allozymes [23] and mitochondrial DNA sequences [24] found conflicting relationships, although both studies were based on limiting sampling. The discrepancies between these studies could be due to discordance between mitochondrial and nuclear gene phylogenies or sampling from different parts of the distribution (e.g., if the allozyme samples were from zones of intergradation). Here we present sequence data for the mitochondrial genes cytochrome *b* (*Cytb*) and *16 s* and a nuclear recombination activating gene-1 (*Rag1*) for *Amphiuma* from across the geographic distribution of the family to estimate lineage diversity. To further reconstruct the relationships among these lineages, we used ∼4 Kb of mitochondrial and ∼3 Kb of nuclear DNA. Using this robust phylogeny for the family and considering ancient fossil amphiumids, we present hypotheses for the evolution of extreme body size changes and its implications for ecological and genetic divergence in this family.

## Methods

### Sampling

Sixty Amphiuma tissue samples were collected from across the distribution of the three currently recognized species or obtained from museum collections (Figure 1, Table S1). The species were preliminarily identified by a combination of their geographic distribution, toe number, and body coloration. All specimens were handled in accordance with Institutional Animal Care and Use Committee (IACUC) protocols at the University of Tulsa, University of California, Berkeley, and the University of Texas at Arlington.

### DNA sequence collection and alignment

DNA was isolated from fresh frozen or ethanol preserved tissues using Qiagen DNeasy extraction kits. To estimate lineage diversity of *Amphiuma* we amplified portions of two mitochondrial genes, *Cytb* (783 bp) and *16 s* (538 bp), and the nuclear gene *Rag1* (825 bp) from specimens from across the distribution of the three species (Figure 1; Table S1). To further test relationships among the major lineages of *Amphiuma*, we amplified portions of the mitochondrial genes cytochrome oxidase-1 (*Co1*, 1260 bp) and NADH dehydrogenase subunit 4 (*Nd4*) and adjacent tRNAs (886 bp), and the nuclear genes *Rag1* (1525 bp), pro-opiomelanocortin (*Pomc*, 481 bp), sodium-calcium exchanger 1 (*Ncx1*, 814 bp), and solute carrier family 8 member 3 (*Slc8a3*, 761 bp). For phylogenetic analyses we used outgroups from three other salamander families: *Ambystoma mexicanum* (Ambystomatidae), *Plethodon cinereus* (Plethodontidae), and *Rhyacotriton variegatus* (Rhyacotritonidae). Most outgroup sequences were taken from Genbank (Tables S1, S2; [25]–[31]), whereas others were collected using the methods described below. The PCR primers used are listed in Table S3 [32]–[35].

PCR products were run on 1% agarose gels, and products of the expected molecular weight were cleaned with either a Millipore PCR_96_ cleanup kit (Montáge™) or ExosapIT (USB Corp). Cycle sequencing reactions using Big Dye v. 3.1 (Applied Biosystems Inc.) were cleaned with Sephadex (Sigma) and sequenced on either an ABI 3730 or 3130xl capillary sequencer. Individual sequences were edited and translated in Sequencher ™ vers 4.8 (Gene Codes Corp). The alignments of protein coding genes were unambiguous, but some length variable regions of the ribosomal gene *16 s* and the tRNAs that were questionable were removed prior to analyses. The lengths of alignments were also trimmed so all sequences in a given alignment were the same length. The final alignment of the geographic variation datasets includes 60 individuals of mtDNA from *Cytb* (651 bp) and *16 s* (377 bp) and 50 individuals of nuclear DNA from *Rag1* (548 bp). For the combined analysis of representative lineages we used a total of 2,948 bp of nuclear DNA from: *Rag1* (1,474 bp), *Pomc* (481 bp), *Ncx1* (735 bp) and *Slc8a3* (258 bp). We also used 4,068 bp of mtDNA from the genes: *Cytb* (779 bp), *16 s* (377 bp), *Co1* (517 bp), *Nd4* (629 bp), *tRNA^HIS^* (54 bp), and also NADH dehydrogenase subunits 1 (*Nd1*, 335 bp) and 2 (*Nd2*, 1,010 bp), and adjacent *tRNAs ^ILE^*, *tRNA^MET^*, *tRNA^TRP^*, *tRNA^ALA^*, *tRNA^ASN^*, and *tRNA^ILE^* (367 bp total) from Genbank [24], [26], [29].

### Phylogenetic analyses and divergence time estimates

Individual genes and datasets including combinations of genes were analyzed with Maximum Parsimony (MP) and Bayesian (BA) methods. Unweighted maximum-parsimony analyses using heuristic searches with 100 random-taxon-addition replicates, and non-parametric bootstrapping [36] based on 1000 pseudoreplicates and 10 random taxon-addition-replicates per pseudoreplicate, were performed in PAUP* v. 4.0b10 [37]. MrModeltest v. 2.2 [38] was used to determine the most appropriate model of nucleotide substitution for each data partition (Table S4). The datasets were partitioned by gene, and protein coding genes were further partitioned by codon position [28], [39], [40]. Alignments including primarily evolutionarily conserved stems of seven tRNAs were combined and analyzed under a single model. Partitioned Bayesian analyses (all partitions unlinked) implemented via MrBayes v. 3.1 [41], [42] were run with four chains (three hot and one cold) and uniform priors for five million generations (with a tree saved at every 1000 generations). We discarded the first one million generations (1000 trees) as burn-in. The resulting 50% majority-rule consensus of the 4001 post burn-in trees, sampled every 100 generations, was computed in PAUP* [37]. By default, MrBayes 3.1 runs each analysis twice simultaneously, and in each case our independent runs converged on the same topology and posterior probabilities for all of the major nodes. We used the Shimodaira-Hasegawa test (SH-test; [43]) implemented in PAUP* [37] to specifically test among the three alternate hypotheses for the relationships of amphiuma: 1. (*A. pholeter* (*A. means*+*A. tridactylum*)); 2. (*A. means* (*A. pholeter*+*A. tridactylum*)); and 3. (*A. tridactylum* (*A. means*+*A. pholeter*)). The SH-test was based on the complete 7 Kb dataset analyzed using GTR+Γ and base frequencies and rate matrix determined by MrModeltest [38].

We estimated divergence times using penalized likelihood (PL) in the program r8s v. 1.7 [44], [45], based on a *Rag1* family-level phylogeny of salamanders with the topology and branch lengths estimated via a partitioned Bayesian analysis in MrBayes [41], [42] (Table S5). The Bayesian analysis of *Rag1* (1,410 bp) was run using four chains (3 hot and 1 cold) for five million generations with a tree saved every 10,000 generations. The first 100 trees (one million generations) were discarded as burnin and the 400 post burnin trees were used to estimate the topology and branch lengths used to estimate the ages of select nodes. The tree was rooted with a caecilian (*Ichthyophis*) and a frog (*Ascaphus montanus*) was also included as an outgroup for the phylogenetic analysis, but both taxa were pruned in r8s prior to calculating divergence times. We fixed the basal split between two major lineages of crown group salamanders (cryptobranchoids and salamandroids) at two different dates: 1) 161 MY, based on the earliest known cryptobranchoid, *Chunerpeton tainyiensis* [46], and 2) 250 MY, which is an approximate average between some of the oldest molecular based divergences for this split which range from 220 to 275 MYA [30], [31], [47]. We also used four fossil salamandroids to serve as minimum external calibration points (Table S6; Figures S1, S2 [48]–[52]). The TN (truncated Newtonian) method was used for PL, and the cross validation procedure was run in eight increments of 0.5 from 0 to 3.5 (on a log10 scale) to test for the optimal smoothing parameter for analyses with the basal node fixed at either 161 or 250 MYA. The optimal smoothing parameters were 32 (161 MY) and 100 (250 MYA). The profile command was used to calculate the mean age and standard deviation for select nodes based on the branch lengths of the 400 post burnin Bayesian trees.

### Analysis of body-size evolution

To assess the extent of body size differences among modern *Amphiuma*, we measured the length and girth of adults from museum collections. Body length was based on measuring both total length (TL = tip of snout to the tip of tail) and snout to vent length (SVL = tip of the snout to the posterior margin of the cloaca), and girth was estimated by measuring the body depth (BD) and body width (BW) immediately anterior to the forelimbs. All specimens measured were at or above the minimum adult body sizes reported for each of the three species: *A. tridactylum*, 33 cm SVL [53]; *A. means*, 26 cm SVL [54]; and *A. pholeter*, 19 cm SVL (based on 24 cm TL [55]). *Amphiuma* are relatively cylindrical in shape, so we estimate the average overall body size (head and trunk) for each species by calculating body volume using the formula for an elliptical cylinder = π×(major axis/2)×(minor axix/2)×Length, where the major axis = BW, minor axis = BD, and length = SVL. Maximum total lengths reported in the literature for these species are: *A. tridactylum*, 106 cm [56], *A. means*, 116 cm [56], [57], and *A. pholeter*, 33 cm [55]. Body lengths of fossil amphiumids and outgroups were taken or estimated from the literature. The small isolated vertebrae of *Proamphiuma cretacea* have well developed crests and heavy ossification, so they are presumed to be from adults estimated to be ∼30 cm TL [49]. Specimens assignable to *Amphiuma jepseni* are limited, but based on its description [48] and the size of the vertebrae, we infer that this specimen is also an adult of small size (∼30 cm TL). Several recent higher-level studies of salamander phylogeny support a clade that includes the families Rhyacotritonidae, Plethodontidae, and Amphiumidae, with strong support for a sister relationship between amphiumids and plethodontids [27], [28], [30]. Rhyacotritonids are not known from the fossil record, and all four extant species are small (adults 7 to 11.5 cm TL; [58]). Similarly, most plethodontid genera comprise relatively small species, and the few “large” species are no longer that *A. pholeter* [20], [59]. Therefore, we consider *A. means* and *A. tridactylum* gigantic species, as they are among the largest extant amphibians, and based on the information above we consider *P. cretacea*, *A. jepseni*, *A. pholeter*, plethodontids, and rhyacotritonids to be small taxa.

Mesquite v 2.5 [60] was used to analyze the ancestral states of the trait maximum body size (TL) as a discrete character (gigantic vs small) using a likelihood framework. This method allows the rate of change between states to be modeled when tracing the evolution of characters on the phylogenetic tree, and calculates the proportional likelihood of the ancestral condition for each node. Our analyses were based on the Markov k-state 1 (Mk1) parameter model that considers an equal rate of change between states. Reconstructions were based on the topology of the 7 Kb molecular dataset with the fossil taxa included based on their taxonomy and distribution in the fossil record (*P. cretacea* (*A. jepseni*+clade based on our estimate of the relationships among extant *Amphiuma*)). One advantage of Maximum Likelihood reconstruction of ancestral states (over parsimony based methods) is the ability to incorporate a time component that is estimated by branch lengths on the phylogeny. We calculated the proportional likelihood of the ancestral condition with and without branch lengths from Bayesian analysis of *Rag1*.

## Results

**Figure 2:**
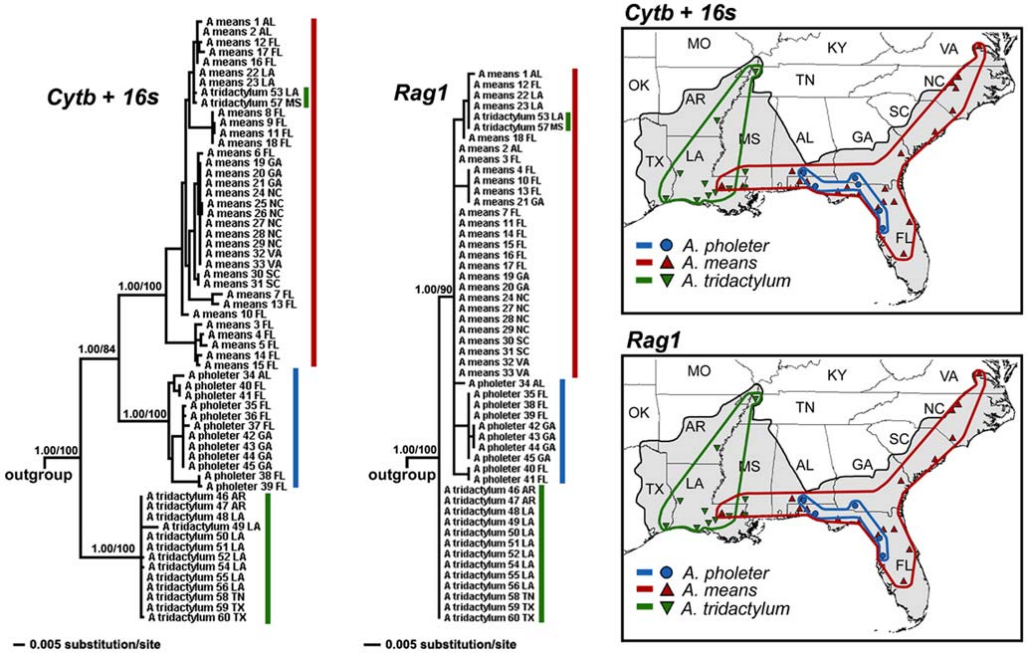
Geographic genetic variation in the Amphiumidae. Bayesian phylograms of the mitochondrial genes *Cytb* and *16 s* (left) and the nuclear gene *Rag1* (right). Numbers subtending the major nodes are Bayesian posterior probabilities to the left of the slash and maximum parsimony bootstrap values to the right. The three major clades, which primarily correspond to the recognized species, are indicated on the phylograms and maps to the right with colored lines: *A. pholeter* (blue), *A. means* (red), and *A. tridactylum* (green). Two putative “*A. tridactylum*” that are closely related to some *A. means* are highlighted on the phylogenies with green triangles.

Geographic variation of mitochondrial DNA (based on *Cytb* and *16 s*) indicates that modern amphiumas contain three divergent genetic lineages that nearly exactly correspond to the three recognized species (Figure 2). Herein we will refer to these lineages with their current taxonomic names. We found almost no variation in mtDNA within *A. tridactylum* (uncorrected P<0.1%) from throughout their distribution. Some variation exists within *A. means* (uncorrected P = 4.37%), primarily within the Gulf Coastal Plain and Florida; populations across the entire Atlantic Coastal Plain from Georgia to Virginia are very similar (uncorrected P<0.5%). We found mitochondrial variation of up to 2.5% among *A. pholeter* from different river drainages. We found little genetic variation in *Rag1* from across the distribution of the three species. However, even with a low level of variation at this locus, *A. means* and *A. pholeter* form a clade, primarily exclusive of *A. tridactylum*. We found no variation in *A. tridactylum* for *Rag1*. There are some nucleotide substitutions among *A. means* and *A. pholeter*, and these species do not form reciprocally monophyletic clades, which may result from incomplete lineage sorting or too little variation to build an accurate tree. We did not find any cases where *A. means* and *A. pholeter* had identical *Rag1* sequences. Two specimens initially identified as *A. tridactylum* (53 and 57) from the Pearl River drainage were identical to *A. means* from the same drainage in both mtDNA and *Rag1*. However, we reexamined specimen 53 and found that it has two toes on some limbs and three toes on others, but it was not heterozygous for any of the otherwise diagnostic nucleotide differences between *A. means* and *A. tridactylum*. Specimen 57 is not available for morphological reexamination, but it had three toes on at least some limbs when collected. We interpret these specimens to be either hybrid backcrosses between *A. means* and *A. tridactylum*, or simply *A. means* with anomalous numbers of toes on some limbs, perhaps a recurrence of the ancestral condition.

**Figure 3:**
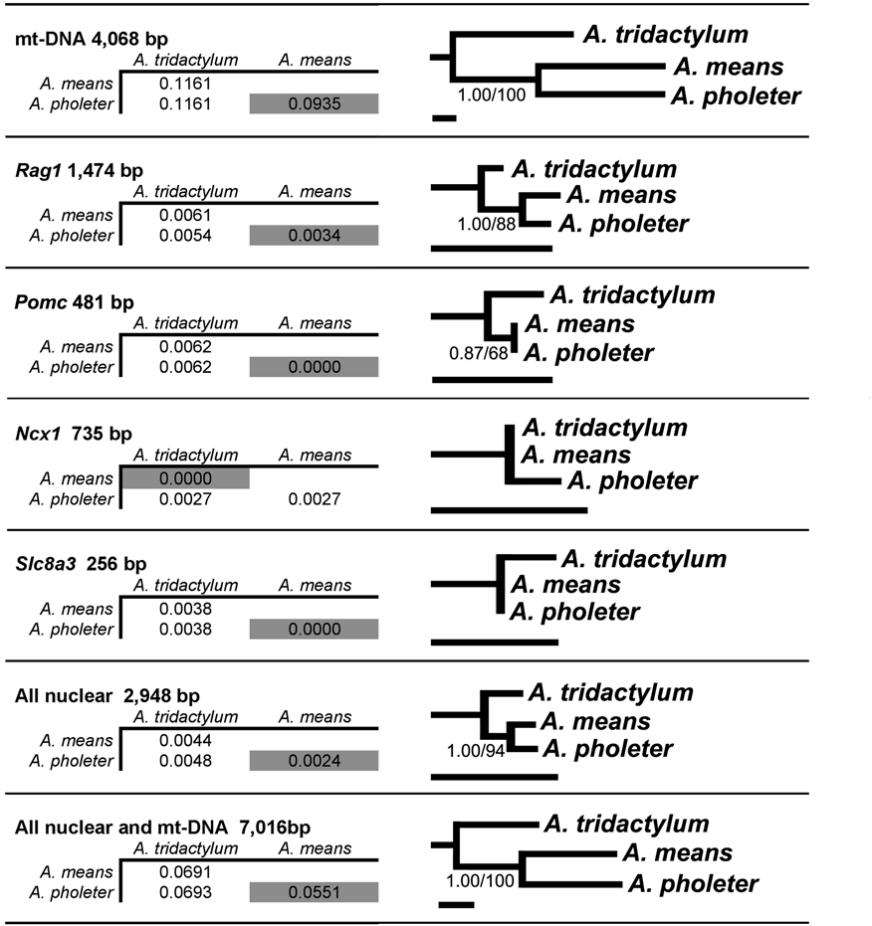
Genetic divergence and phylogenetic relationships for the nominate taxa of *Amphiuma* based on mitochondrial and nuclear genes. Matrices to the left are uncorrected pairwise sequence divergence among the three taxa with the most closely related pair highlighted in grey. Bayesian phylograms on the right describe the relationships among the three taxa, and statistical support, if any, is indicated by Bayesian posterior probabilities to the left of the slash and maximum parsimony bootstrap values to the right.

The uncorrected pairwise divergence of mitochondrial genes and three of the four nuclear loci show *A. tridactylum* to be the most divergent lineage of *Amphiuma*, whereas *A. means* and *A. pholeter* are most similar (Figure 3). Only the nuclear gene *Ncx1* shows *A. pholeter* to be the most divergent lineage of *Amphiuma*, but that is based upon only a single substitution in *A. pholeter* out of 735 bp; the three species are otherwise identical. Phylogenetic analyses show strong support for *A. means* and *A. pholeter* as a clade exclusive of *A. tridactylum*, based on mitochondrial DNA alone (BAPP = 1.00, MPBS = 100), the combined nuclear data (BAPP = 1.00, MPBS = 94), and the combined mitochondrial and nuclear data (BAPP = 1.00, MPBS = 100). Analyses of each nuclear gene alone support either the *A. means*+*A. pholeter* clade (*Rag1* and *Pomc*) or were unable to resolve the relationships among the lineages (*Ncx1* and *Slc8a3*). Maximum likelihood analyses based on the combined dataset provided strong support for an *A. means*+*A. pholeter* clade (MLBS = 100). Furthermore, SH-tests show this topology to be significantly more likely than the ML phylogeny with *A. means* and *A. tridactylum*, or *A. pholeter* and *A. tridactylum*, constrained to be monophyletic (Table 1). Means and Karlin's [23] genetically similar samples of *A. means* and *A. tridactylum* fall well within our genetically divergent geographic lineages of *A. means* and *A. tridactylum* for both mt-DNA and *Rag1*. Although we find very low levels of nuclear variation in *Amphiuma*, it is peculiar that they found *A. pholeter* to be so divergent from *A. means* and *A. tridactylum*. The only way that we can reconcile this discrepancy is if all of Means and Karlin's [23] allozyme loci are geographically discordant with *Rag1* and mitochondrial variation, or if they have had increased rates of evolution in the *A. pholeter* lineage.

**Table 1:** Results of Shimodaira-Hasegawa tests of two constrained alternate topologies to an unconstrained maximum likelihood analysis based on the combined mitochondrial and nuclear dataset.

test	topology	−*ln* likelihood	Difference in −*ln* likelihood	*p*
	Unconstrained *A. means* and *A. pholeter* monophyletic	24811.97	----	----
**1**	*A. means* and *A. tridactylum* monophyletic	24845.88	33.91	<0.001
**2**	*A. pholeter* and *A. tridactylum* monophyletic	24847.29	35.32	<0.001

Divergence time estimates based on penalized likelihood of *Rag1* show a most recent common ancestor of modern *Amphiuma* (i.e., the split between *A. tridactylum* and *A. means*+*A. pholeter*) to be 5.0±1.5 MYA or 7.8±2.3 MYA, and divergence between *A. means* and *A. pholeter* to be 2.1±0.8 MYA or 3.2±1.2 MYA. Theses alternate dates for each node represent analyses based on fixing the basal node (cryptobranchoids+salamandroids) at either 161 MYA [46] or 250 MYA [30], [31], [47] respectively (See Methods; Tables 2, S6; Figures S1, S2).

**Table 2:** Results of divergence time estimates (Average±Standard deviation) based on a Baysian *Rag1* phylogeny of salamanders.

Node	PL (161)	PL (250)
*A. means*+*A. pholeter*	2.1±0.8	3.2±1.2
*A. tridactylum*+*A. means*+*A. pholeter*	5.0±1.5	7.8±2.3
Plethodontidae+Amphiumidae	78.1±7.6	121.8±12.0
Rhyacotritonidae+Plethodontidae+Amphiumidae	98.4±9.1	153.0±14.2

Dates were estimated using penalized likelihood (PL) and fixing the basal split between cryptobranchoids and salamandroids at either 161 MY or 250 MY.

**Figure 4:**
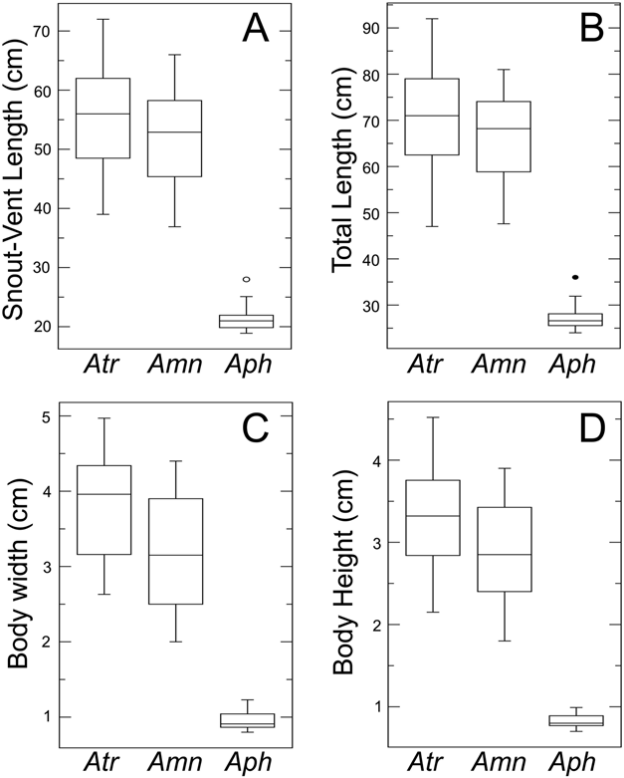
Body sizes of adult *Amphiuma*. Box plots of A) snout-vent length, B) total length, C) body width, and D) body height for *A. tridactylum* (Atr), *A. means* (Amn), and *A. pholeter* (Aph) measured in this study. The five horizontal lines of each plot represent the minimum, first quartile, median, third quartile, and maximum values for each species. Filled circles represent outliers and open circles represent suspected outliers. Averages, and ranges for each measurement and species are listed in Table 3.

**Figure 5:**
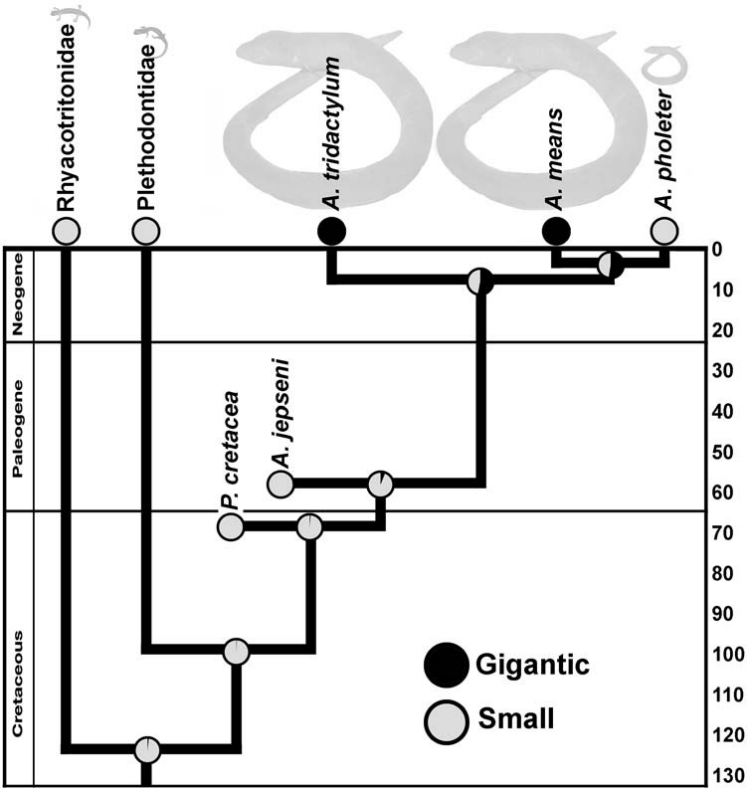
Divergence time estimates and body-size evolution of amphiumid salamanders. Extant taxa on the chronogram are drawn half way between the results of the two different penalized likelihood analysis of *Rag1* from r8s (Table 2). Extinct amphiumids were added to the tree based on their distribution in the fossil record and suggested relationships [49]. Reconstruction of ancestral body size (gigantic vs small) was performed using a maximum likelihood algorithm in Mesquite. Pie diagrams at each node indicate the likelihood of each state. The ancestral state data shown are based on analyses without branch lengths (Table 4).

Our body size and girth measurements show that average adult *A. means* and *A. tridactylum* are about 2.5 times longer, and ∼3.5 to 4 times wider and deeper than *A. pholeter* (Table 3; Figure 4). Taken together the gigantic species are on average >30 (*A. means*) and 45 (*A. tridactylum*) times larger (in volume) than *A. pholeter* (Table 3). This difference is the same whether we estimate overall body size (head and trunk) as an elliptical cylinder (π×(BW/2)×(BD/2)×SVL) or as a rectangular prism (BW×BD×SVL). Maximum likelihood reconstruction of ancestral body size as a discrete character (gigantic vs small), assuming an equal rate of change between states, and considering extant and fossil taxa shows a marginally higher proportional likelihood for gigantism as the ancestral condition for modern *Amphiuma* (0.530) and also the ancestor of the clade *A. means*+*A. pholeter* (0.524; Table 4, Figure 5). The proportional likelihood for gigantism increases slightly for both modern *Amphiuma* (0.576) and also the ancestor of the clade *A. means*+*A. pholeter* (0.567) when *Rag1* branch length information is incorporated in the calculation (Table 4).

**Table 3:** *Amphiuma* body-size.

Species	n	SVL	TL	BW	BD	Head and trunk volume π×(BW/2)×(BD/2)×SVL
***A. tridactylum***	25	55.4 (39.0–72.0)	70.5 (47.0–92.0)	3.8 (2.6–5.0)	3.3 (2.2–4.5)	545.6 (195.3–1128.6)
***A. means***	18	52.0 (36.9–66.0)	66.6 (47.6–81.0)	3.2 (2.0–4.4)	2.9 (1.8–3.9)	379.0 (99.3–812.2)
***A. pholeter***	12	21.4 (18.9–28.0)	27.4 (24.0–36.0)	0.9 (0.8–1.2)	0.8 (0.7–1.0)	12.1 (9.6–25)

Numbers in parenthesis represent upper and lower values for each measurement and the number above is the average. Measurements are in centimeters (cm) and volume estimate is in cm^3^.

**Table 4:** Results of maximum likelihood ancestral state reconstruction of body-size, of extant *Amphiuma*, fossil amphiumids, and closely related families.

Node description	Likelihood Small	Likelihood Gigantic
**Without Branch lengths**		
*A. means*+*A. pholeter*	0.476	0.524
*A. tridactylum*+*A. means*+*A. pholeter*	0.470	0.530
*Amphiuma*	0.939	0.061
Amphiumidae	0.991	0.009
Plethodontidae+Amphiumidae	0.996	0.004
Rhyacotritonidae+Plethodontidae+Amphiumidae	0.985	0.015
**With Branch lengths**		
*A. means*+*A. pholeter*	0.433	0.567
*A. tridactylum*+*A. means*+*A. pholeter*	0.424	0.576
*Amphiuma*	0.987	0.013
Amphiumidae	0.997	0.003
Plethodontidae+Amphiumidae	0.959	0.041
Rhyacotritonidae+Plethodontidae+Amphiumidae	0.936	0.061

Analyses were run with and without Bayesian branch lengths. Both analyses were based on Mk1 model that assumes an equal rate of transition between the two states (small vs. gigantic).

## Discussion

### Phylogeny and evolution of body-size in the Amphiumidae

The earliest molecular systematic study of all species of *Amphiuma*, based on allozymes, found the gigantic species, *A. means* and *A. tridactylum*, to be closely related (Nei's D = 0.12), whereas, *A. pholeter* is very divergent (Nei's D = 0.90 from *A. means* and 0.73 from *A. tridactylum*) and represented an “ancient evolutionary offshoot” [23]. A more recent phylogentic analysis of salamander families based on mitochondrial DNA sequences included an individual of all three species of *Amphiuma* and showed *A. means* and *A. pholeter* to be sister taxa [24]. Our analysis based on widespread sampling of both nuclear and mitochondrial DNA sequences across the geographic distribution of the Amphiumidae, reveals three primary genetic lineages that correspond to the three recognized species (Figure 2). Consistent with the second study [24], we found strong support for a sister relationship between *A. means* and *A. pholeter* (Figure 3, Table 1). Furthermore, our divergence time estimates indicate that *A tridactylum* represents the earliest diverged lineage among modern species, whereas *A. means* and *A. pholeter* share a more recent common ancestor. We estimate the oldest divergence among modern lineages of *Amphiuma* to be no older than the Miocene, suggesting that the two definitive fossil amphiumids from the Upper Cretaceous (*Proamphiuma cretacea*) and Paleocene (*Amphiuma jepseni*) [52], [53] are indeed outgroups. In summary our hypothesis for the relationships of the family Amphiumidae are: (*P. cretacea* (*A. jepseni* (*A. tridactylum* (*A. means*+*A. pholeter*)))).

Closely related families of salamanders (rhyacotritonids and plethodontids), fossil amphiumids from the Upper Cretaceous (*Proamphiuma cretacea*) and Paleocene (*Amphiuma jepseni*), and *A. pholeter* are small, whereas *A. means* and *A. tridactylum* are gigantic (>30 to 45 times larger than *A. pholeter*). Therefore, our phylogeny and reconstruction suggest two possible scenarios for the evolution of gigantism in this family: 1) Gigantic body size either evolved once, since the Paleocene, and was the ancestral condition of modern amphiumas, with a subsequent strong size reduction in *A. pholeter* or 2) small body size was the ancestral condition of extant *Amphiuma* and gigantism independently evolved in the lineages leading to the two modern species *A. tridactylum* and *A. means*. Maximum likelihood reconstruction shows a marginally higher proportional likelihood for gigantism as the ancestral condition for modern *Amphiuma* and also for the most recent common ancestor of *A. means* and *A. pholeter* (Figure 5). Given that our analysis of widespread geographic genetic variation revealed only three genetic lineages of modern amphiumids, our ability of further address the evolution of body-size by examining modern species is limited. However, the discovery of additional fossil lineages would greatly enhance our understanding of the evolution of body size of amphiumid salamanders. *Amphiuma antica* [61] was described from the mid-Miocene of Texas based on a single large, poorly preserved vertebra, but the assignment of this specimen to the Amphiumidae is questionable [49]. Gardner [49] also suggested that it could be one of the modern species of *Amphiuma*. We estimate that modern *Amphiuma* share a common ancestor in the late Miocene. If *A. antica* is gigantic *Amphiuma* and a stem or sister taxon to modern species, then this would strongly support our first hypothesis that the ancestor of modern *Amphiuma* was gigantic and the relatively small *A. pholeter* results from miniaturization. Regardless of the direction, or the number of times body size has changed in the Amphiumidae, this extreme change has happened over a relatively short period of time, since the sister taxa, *A. means* and *A. pholeter*, shared a common ancestor as recently as the late-Pliocene.

### Niche breadth, distribution size, and reproductive isolation

Body size is a key parameter for determining the relative placement of an organism in its environment and can also impact its niche breadth, dispersal ability, and consequently, geographic distribution [6]–[9]. The extreme difference in body size among modern *Amphiuma* is coincident with strong differences in niche breadth and geographic distribution. The gigantic species, *A. means* and *A. tridactylum*, are widespread species that occur in diverse lowland aquatic environments, including swamps, ponds, marshes, rivers, and drainage ditches [20], [62], [63]. In contrast, the small species, *A. pholeter* has a relatively limited distribution and is restricted to specific organic muck (fine mud) habitats [55], [64]. Our first scenario for the evolution of body size in the Amphiumidae suggests that a habitat specialist evolved via miniturization of a gigantic, habitat generalist. The organic muck habitats where *A. pholeter* occur are derived from finely decayed plant matter that builds up as deep beds in lowland aquatic habitats. Interestingly, only juvenile *A. means* have been found syntopicly with *A. pholeter* in the muck habitats [64; RWV and PEM, pers. obs.], so this unique habitat may only be favorable for small *Amphiuma*.

The estimated age for sexual maturity for *A. means* is 3 to 4 years (26 cm SVL) [65], whereas for *A. pholeter* it is only 2 years (19 cm SVL) or less [20]. Therefore, the miniature body size of *A. pholeter* may have occurred by early maturation (of a gigantic ancestor) during the evolution of a completely muck-dwelling existence. This semi-liquid muck appears to provide a substantial amount of support to the bodies of *A. pholeter*, as their locomotor ability is relatively limited in open water (RWV and PEM, pers. obs.). Even though fine muck habitats occur throughout the Coastal Plain, the relatively restricted current distribution of *A. pholeter* may result from geographic barriers, such as ridges and large rivers that limit their dispersal between muck habitats.

If scenario two is correct and recent ancestors of modern *Amphiuma* were small, two independent instances of gigantism are likely in the *A. means* and *A. tridactylum* lineages. This scenario implies parallel instances of Cope's Rule (evolutionary increase in body-size), where delaying maturation and drastically increasing overall body size would have had strong fitness consequences such as fecundity and survival. The Coastal Plain of the southeastern United States includes a wide range of lowland aquatic habitats. Gigantic body size may further allow *A. means* and *A. tridactylum* to traverse and colonize the wide breadth of habitats that occur across the Coastal Plain.

Evolution of body size is a simple mechanism for generating ecological and genetic divergence [1]–[5]. The shift in habitat use appears to be a distinct partition between gigantic and small *Amphiuma*, because *A. pholeter* spends almost its entire life in a specialized habitat that is not commonly utilized by the adults of *A. means*. This shift in habitat and body size may have provided a strong barrier for promoting genetic divergence between these species. Previous morphological analysis of the two gigantic species, *A. means* and *A. tridactylum*, from across their zone of overlap in the mid-Gulf Coastal Plain found them to be distinct species, but identified putative hybrid individuals from the Pearl River drainage that had an amalgam of otherwise species specific traits, including specimens with two toes on some limbs and three toes on others [21]. Our samples from this region all had mitochondrial haplotypes and *Rag1* alleles similar to those of *A. means*, even though some had three toes on some limbs. In contrast, the distribution of the small species, *A. pholeter*, is entirely within that of one of the gigantic species, *A. means*. Despite the fact that they are the most closely related species of modern *Amphiuma*, these species, so strongly divergent in size, are not known to interbreed. More detailed genetic sampling along the contact zone is necessary to further test whether *A. means* and *A. tridactylum* hybridize. Also, more detailed sampling of microsympatric populations of *A. means* and *A. pholeter* would test if there has been any recent genetic interaction. If *A. means* and *A. tridactylum* interbreed but *A. means* and *A. pholeter* do not, body size and habitat specialization may serve as a greater isolating mechanism than genetic divergence alone.

In conclusion, our phylogeograpghic analyses based on both mitochondrial and nuclear DNA indicate three divergent lineages of modern *Amphiuma* that closely correspond to the three currently recognized species. Nearly all molecular data support a sister relationship between *A. means* and *A. pholeter* which diverged as recently as the late-Pliocene. When placing this relationship in the context of fossil amphiumids and closely related families we find that there has been either: 1) a single case of gigantism in the common ancestor of modern *Amphiuma* and subsequently a recent instance of miniaturization in *A. pholeter*, possibly as a specialization to a completely muck-dwelling existence, or 2) two independent instances of gigantism in *A. means* and *A. tridactylum*, which may contribute to their ability to traverse and inhabit a wide variety of lowland aquatic habitats. Additional fossils of mid-Cenozoic amphiumids will greatly enhance our understanding of the direction of body size evolution in these salamanders.

## Supporting Information

Table S1 Specimen information and Genbank numbers 16 s, Cytb, and Rag1 from across the distribution of all three species of Amphiuma.
(0.07 MB DOC)

Table S2 Specimen information and Genbank numbers for Amphiuma and outgroups used for individual and combined analyses of mitochondrial and nuclear loci (Figure 3). 16 s, Cytb, and Rag1 for these analyses are listed in Table S1.
(0.04 MB DOC)

Table S3 Primers used for PCR and sequencing.
(0.05 MB DOC)

Table S4 Models applied to each data partition for Bayesian analyses.
(0.05 MB DOC)

Table S5 Species and Genbank numbers for Bayesian phylogenetic analysis of Rag1 that was used for divergence time estimates in r8s.
(0.04 MB DOC)

Table S6 External calibration points used for nonparametric rate smoothing analysis of Rag1 using r8s. Points are plotted on Figures S1 and S2.
(0.04 MB DOC)

Figure S1 Chronogram of salamander families calculated in r8s based on Bayesian analysis of Rag1, fixing the basal node at 161 MYA, and four external calibration points (Table S5, S6).
(5.76 MB TIF)

Figure S2 Chronogram of salamander families calculated in r8s based on Bayesian analysis of Rag1, fixing the basal node at 250 MYA, and four external calibration points (Table S5, S6).
(5.73 MB TIF)
