# Supplementary material for: Evolution of Gigantism in Amphiumid Salamanders
Source: PLoS One. 2009 May 20;4(5):e5615. doi: 10.1371/journal.pone.0005615 (PMC2680017; doi:10.1371/journal.pone.0005615)
Supplement: Table S1 — Specimen information and Genbank numbers 16 s, Cytb, and Rag1 from across the distribution of all three species of Amphiuma. (0.07 MB DOC) [file pone.0005615.s001.doc]

| **Tree code/ map number** | **Locality**  **State: County, location** | **Museum/**  **Field**  **Number** | **Genbank**  **Accession *16s*** | **Genbank**  **Accession *Cytb*** | **Genbank**  **Accession *Rag1*** |  | | | |
| --- | --- | --- | --- | --- | --- | --- | --- | --- | --- |
| ***Amphiuma means* (two-toed amphiuma):** | | | | | |  | | | |
| 1 | AL: Covington, Pond Creek Bridge | AUM 37420 | FJ951240 | FJ951304 | FJ951370 |  | | | |
| 2 | AL: Escambia, Conecuh N.F. | AUM 37447 | FJ951241 | FJ951305 | FJ951371 |  | | | |
| 3 | FL: Alachua, River Styx | ASU 24181 | FJ951242 | FJ951306 | FJ951372 |  | | | |
| 4 | FL: Alachua, Gainesville | RMB 3005 | FJ951243 | FJ951307 | FJ951373 |  | | | |
| 5 | FL: Alachua, Orange Lake | MVZ 144889 | FJ951244 | FJ951308 | - |  | | | |
| 6 | FL: Baker, Oceola N.F. Forest Rd. 241 | MVZ 241516 | FJ951245 | FJ951309 | - |  | | | |
| 7 | FL: Glades, Pollywog Creek | UF 135378 | FJ951246 | FJ951310 | FJ951374 |  | | | |
| 8 | FL: Leon, Clear Lake | MVZ 241517 | FJ951247 | FJ951311 | - |  | | | |
| 9 | FL: Leon, Silver Lake | MVZ 241518 | FJ951248 | FJ951312 | - |  | | | |
| 10 | FL: Levy, Gulf Hammock | APPSU 23768 | FJ951249 | AY691722 | AY650127 |  | | | |
| 11 | FL: Liberty, Ochlockonee River | RMB 2983 | FJ951250 | FJ951313 | FJ951375 |  | | | |
| 12 | FL: Okaloosa, Walton Pond | ASU 24552 | FJ951251 | FJ951314 | FJ951376 |  | | | |
| 13 | FL: Orange, Shingle Creek | RMB 3006 | FJ951252 | FJ951315 | FJ951377 |  | | | |
| 14 | FL: Putnam, Dunns Creek State Park | UF133757 | FJ951253 | FJ951316 | FJ951378 |  | | | |
| 15 | FL: Putnam, Rodman Reservoir | RMB 3007 | FJ951254 | FJ951317 | FJ951379 |  | | | |
| 16 | FL: Santa Rosa, Escambia River | ASU 25020 | FJ951255 | FJ951318 | FJ951380 |  | | | |
| 17 | FL: Santa Rosa, near Munson | ASU 24554 | FJ951256 | FJ951319 | FJ951381 |  | | | |
| 18 | FL: Taylor, US 27, 4 mi S of Madison Co | RMB 3001 | FJ951257 | FJ951320 | FJ951382 |  | | | |
| 19 | GA: Liberty, near Hinesville | RMB 3131 | FJ951258 | FJ951321 | FJ951383 |  | | | |
| 20 | GA: Liberty, near Hinesville | RMB 3132 | FJ951259 | FJ951322 | FJ951384 |  | | | |
| 21 | GA: Liberty, near Hinesville | RMB 3133 | FJ951260 | FJ951323 | FJ951385 |  | | | |
| 22 | LA: St. Tammany, Talisheek Bay | LSUMZ H-3333 | FJ951261 | FJ951324 | FJ951386 |  | | | |
| 23 | LA: East Baton Rouge, Camp Istrouma | LSUMZ H-15033 | FJ951262 | FJ951325 | FJ951387 |  | | | |
| 24 | NC: Columbus, Lake Waccamaw | RMB 3251 | FJ951263 | FJ951326 | FJ951388 |  | | | |
| 25 | NC: Johnston, Clayton | MVZ 137236 | FJ951264 | FJ951327 | - |  | | | |
| 26 | NC: Johnston, Clayton | MVZ 137237 | FJ951265 | FJ951328 | - |  | | | |
| 27 | NC: Nash, near Stenhope | RMB 3187 | FJ951266 | FJ951329 | FJ951389 |  | | | |
| 28 | NC: Wake, near Garner | RMB 3134 | FJ951267 | FJ951330 | FJ951390 |  | | | |
| 29 | NC: Wake, near Garner | RMB 3135 | FJ951268 | FJ951331 | FJ951391 |  | | | |
| 30 | SC: Berkeley, Francis Marion NF | RMB 3254 | FJ951269 | FJ951332 | FJ951392 |  | | | |
| 31 | SC: Berkeley, Francis Marion NF | ASU 24509 | FJ951270 | FJ951333 | FJ951393 |  | | | |
| 32 | VA: Charles City, Charles City | RMB 3249 | FJ951271 | FJ951334 | FJ951394 |  | | | |
| 33 | VA: Charles City, Charles City | RMB 3250 | FJ951272 | FJ951335 | FJ951395 |  | | | |
| ***Amphiuma pholeter* (one-toed amphiuma):** | | | | | |  | | | |
| 34 | AL: Covington, Pond Creek | AUM 37412 | FJ951273 | FJ951336 | FJ951396 |  | | | |
| 35 | FL: Hernando, Chassahowitzka WMA | RMB 3010 | FJ951274 | FJ951337 | FJ951397 |  | | | |
| 36 | FL: Jefferson, near Capps | ASU 23947 | FJ951275 | FJ951338 | - |  | | | |
| 37 | FL: Jefferson, near Capps | ASU 23948 | FJ951276 | FJ951339 | - |  | | | |
| 38 | FL: Levy, Gulf Hammock | RMB 3255 | FJ951277 | FJ951340 | FJ951398 |  | | | |
| 39 | FL: Levy, Gulf Hammock | APPSU 23767 | FJ951278 | AY691766 | AY650128 |  | | | |
| 40 | FL: Walton, near Freeport | ASU 24330 | FJ951279 | FJ951341 | FJ951399 |  | | | |
| 41 | FL: Walton, near Freeport | ASU 23914 | FJ951280 | FJ951342 | FJ951400 |  | | | |
| 42 | GA: Grady, Ochlockonee River | RMB 3256 | FJ951281 | FJ951343 | FJ951401 |  | | | |
| 43 | GA: Grady, Ochlockonee River | RMB 3257 | FJ951282 | FJ951344 | FJ951402 |  | | | |
| 44 | GA: Grady, Ochlockonee River | RMB 3258 | FJ951283 | FJ951345 | FJ951403 |  | | | |
| 45 | GA: Grady, Ochlockonee River | RMB 3259 | FJ951284 | FJ951346 | FJ951404 |  | | | |
| ***Amphiuma tridactylum* (three-toed amphiuma):** | | | | | |  | | | |
| 46 | AR: Desha, T10S, R2W, S25 | ASUMZ 29092 | FJ951285 | FJ951347 | FJ951405 |  | | | |
| 47 | AR: Desha, T10S, R2W, S25 | ASUMZ 29093 | FJ951286 | FJ951348 | FJ951406 |  | | | |
| 48 | LA: Iberville, near Gabriel | LSUMZ H-877 | FJ951287 | FJ951349 | FJ951407 |  | | | |
| 49 | LA: Livingston, ~2 mi NE of Watson | LSUMZ H-2793 | FJ951288 | FJ951350 | FJ951408 |  | | | |
| 50 | LA: St. Landry, I-49, ~2 mi W of Whiteville | RMB 3093 | FJ951289 | FJ951351 | FJ951409 |  | | | |
| 51 | LA: St. Martin, St. Martinville | BRM 1055 | FJ951290 | FJ951352 | FJ951410 |  | | | |
| 52 | LA: St. Martin, St. Martinville | BRM 1056 | FJ951291 | FJ951353 | FJ951411 |  | | | |
| 53 | LA: St. Tammany, Talisheek Bay | LSUMZ H-3334 | FJ951292 | FJ951354 | FJ951412 |  | | | |
| 54 | LA: Vermillion, ~5 km NW of Andrew | BRM 1075 | FJ951293 | FJ951355 | FJ951413 |  | | | |
| 55 | LA: Vermillion, ~5 km NW of Andrew | BRM 1076 | FJ951294 | FJ951355 | FJ951414 |  | | | |
| 56 | LA: Vernon, Kisatchie NF | MVZ 241480 | FJ951295 | FJ951356 | FJ951415 |  | | | |
| 57 | MS: Pearl River | ASU 10218 | FJ951296 | FJ951357 | FJ951416 |  | | | |
| 58 | TN: Lake or Obion, Reel Foot Lake | RMB 3136 | FJ951297 | FJ951358 | FJ951417 |  | | | |
| 59 | TX: Jefferson, Port Arthur | RMB 3253 | FJ951298 | FJ951359 | FJ951369 |  | | | |
| 60 | TX: Jefferson, Port Arthur | RMB 2982 | FJ951299 | FJ951360 | FJ951418 |  | | | |
| Outgroups: | | | | | |  |  |  | FJ951350 |
| Plethodontidae*: Plethodon cinereus* | | | AY728232 | AY728232 | AY691745 |  | | | |
| Rhyacotritonidae: *Rhyacotriton variegatus* | | | AY728219 | AY728219 | AY691693 |  | | | |
| Ambystomatidae: *Ambystoma mexicanum* | | | AY659991 | AY659991 | EF107293 |  | | | |

APPSU = Appalachian State University

ASUMZ = Arkansas State University Museum of Zoology

AUM = Auburn University Natural History Museum

BRM = Brad R. Moon field series

LSUMZ = Museum of Natural Science, Louisiana State University

MVZ = Museum of Vertebrate Zoology, University of California, Berkeley

RMB = Ronald M. Bonett field series

UF = Florida Museum of Natural History, University of Florida

UTA = Reptile Diversity Research Center, University of Texas at Arlington

- = no sequence collected for the individual
