# Supplementary material for: Evolution of Gigantism in Amphiumid Salamanders
Source: PLoS One. 2009 May 20;4(5):e5615. doi: 10.1371/journal.pone.0005615 (PMC2680017; doi:10.1371/journal.pone.0005615)
Supplement: Table S2 — Specimen information and Genbank numbers for Amphiuma and outgroups used for individual and combined analyses of mitochondrial and nuclear loci (Figure 3). 16 s, Cytb, and Rag1 for these analyses are listed in Table S1. (0.04 MB DOC) [file pone.0005615.s002.doc]

| **Family:**  **Species** | **Genbank**  **Accession**  ***Pomc*** | **Genbank**  **Accession**  ***Ncx1*** | **Genbank**  **Accession *Slc8a3*** | **Genbank**  **Accession *Nd1, ND2,* & tRNAs** | **Genbank**  **Accession**  ***Cox1*** | **Genbank**  **Accession *Nd4* & tRNAs** |
| --- | --- | --- | --- | --- | --- | --- |
| **Ambystomatidae:**  *Ambystoma mexicanum* | EU275841* | EF107230 | EF107367 | AY659991 | AY659991 | AY659991 |
| **Rhyacotritonidae:**  *Rhyacotriton variegatus* | EU275823 | EF107242 | EF107401 | AY728219 | AY728219 | AY728219 |
| **Plethodontidae:**  *Plethodon cinereus* | FJ951365 | EF107226 | EF107360 | AY728232 | AY728232 | AY728232 |
| **Amphiumidae:**  *Amphiuma means* | FJ951366 | FJ951362 | FJ951420 | AY916037 | FJ951301 | AY691766 |
| *Amphiuma pholeter* | FJ951367 | FJ951363 | FJ951421 | AY916035 | FJ951302 | AY691767 |
| *Amphiuma tridactylum* | FJ951368 | FJ951361 | FJ951419 | AY916036 | FJ951300 | FJ951364 |

* *Ambystoma rosaceum*
