# Supplementary material for: Evolution of Gigantism in Amphiumid Salamanders
Source: PLoS One. 2009 May 20;4(5):e5615. doi: 10.1371/journal.pone.0005615 (PMC2680017; doi:10.1371/journal.pone.0005615)
Supplement: Table S3 — Primers used for PCR and sequencing. (0.05 MB DOC) [file pone.0005615.s003.doc]

| **Gene** | **Primer Name** | **Primer sequence** | **Size* (bp)** | **Reference** |
| --- | --- | --- | --- | --- |
| *16s* | 16Sa-L | 5'-CGCCTGTTTATCAAAAACAT-3' | 538 | [32] |
|  | 16Sb-H | 5'-CCGGTCTGAACTCAGATCACGT-3' |  |  |
| *Cytb* | MVZ15 | 5’-GAACTAATGGCCCACACWWTACGNAA-3’ | 783 | [33] |
|  | MVZ16 | 5’-AAATAGGAARTATCAYTCTGGTTTRAT-3’ |  |  |
| *Nd4* | ND4F | 5’-CACCTATGACTACCAAAAGCTCATGTAGAAGC-3’ | 885 | [34] |
|  | LeuR | 5’-CATTACTTTTACTTGGATTTGCACCA-3’ |  |  |
| *Co1* | MVZ_201 | 5’-TCAACAAAYCATAAAGATATTGGCACC-3’ | 1260 | [35] |
|  | MVZ_202 | 5’-GCGTCWGGGTARTCTGAATATCGTCG-3’ |  |  |
| *Rag1* | AmphRag1F | 5’-TGCCCGGCTATCATCCCTTTGAAT–3’ | 825 | This study |
|  | AmphRag1R | 5’-ACCTCCCTCACAAGCTTCTCATCA–3’ |  |  |
| *Pomc* | POMC_Amphiu_F | 5’-ATATGTCATGAGCCATTTTCGCTGGAA-3’ | 481 | This study |
|  | POMC_Amphiu_R | 5’-GGCATTTTTGAAAAGAGTCATTAGAGG-3’ |  |  |
| *Ncx1* | NCX1_Amphiu_A_F | 5’–GCACTATTGTTGGAAGTGCTGCCT-3’ | 814 | This study |
|  | NCX1_Amphiu_A_R | 5’–TCAGATCCTGCATTAGCTGTGCCA-3’ |  |  |
| *Slc8a3* | SLC8A3_Amphiu_A_F | 5’–TGCGGTCATGGGTTTATAGCTGGA-3’ | 761 | This study |
|  | SLC8A3_Amphiu_A_R | 5’–ATGTCACCACCCTTACGAACCACA-3’ |  |  |

Ambiguity codes: N = G, A, T or C; R = A or G; W = A or T; Y = C or T.

* Fragment size between the primer pairs for each gene.
