# Supplementary material for: Evolution of Gigantism in Amphiumid Salamanders
Source: PLoS One. 2009 May 20;4(5):e5615. doi: 10.1371/journal.pone.0005615 (PMC2680017; doi:10.1371/journal.pone.0005615)
Supplement: Table S4 — Models applied to each data partition for Bayesian analyses. (0.05 MB DOC) [file pone.0005615.s004.doc]

| **Gene/partition** | **# of positions in partition** | | **nst** | **rates** | **statefreqpr** |
| --- | --- | --- | --- | --- | --- |
| *Rag1*: alignment pos 1, codon pos 1 | | 492 | 6 | gamma | dirichlet (1,1,1,1) |
| *Rag1*: alignment pos 2, codon pos 2 | | 491 | 1 | equal | dirichlet (1,1,1,1) |
| *Rag1*: alignment pos 3, codon pos 3 | | 491 | 6 | gamma | dirichlet (1,1,1,1) |
| *Pomc*: alignment pos 1, codon pos 3 | | 161 | 6 | gamma | dirichlet (1,1,1,1) |
| *Pomc*: alignment pos 2, codon pos 1 | | 160 | 2 | equal | dirichlet (1,1,1,1) |
| *Pomc*: alignment pos 3, codon pos 2 | | 160 | 6 | equal | dirichlet (1,1,1,1) |
| *Ncx1*: alignment pos 1, codon pos 3 | | 245 | 6 | equal | fixed (equal) |
| *Ncx1*: alignment pos 2, codon pos 1 | | 245 | 6 | propinv | dirichlet (1,1,1,1) |
| *Ncx1*: alignment pos 3, codon pos 2 | | 245 | 1 | equal | dirichlet (1,1,1,1) |
| *Slc8a3*: alignment pos 1, codon pos 2 | | 86 | 1 | equal | dirichlet (1,1,1,1) |
| *Slc8a3*: alignment pos 2, codon pos 3 | | 86 | 2 | equal | dirichlet (1,1,1,1) |
| *Slc8a3*: alignment pos 3, codon pos 1 | | 86 | 1 | equal | dirichlet (1,1,1,1) |
| *16s* | | 377 | 6 | propinv | dirichlet (1,1,1,1) |
| *Nad1*: alignment pos 1, codon pos 1 | | 112 | 2 | propinv | dirichlet (1,1,1,1) |
| *Nad1*: alignment pos 2, codon pos 2 | | 112 | 2 | propinv | dirichlet (1,1,1,1) |
| *Nad1*: alignment pos 3, codon pos 3 | | 111 | 2 | equal | dirichlet (1,1,1,1) |
| *Nad2*: alignment pos 1, codon pos 1 | | 337 | 6 | gamma | dirichlet (1,1,1,1) |
| *Nad2*: alignment pos 2, codon pos 2 | | 337 | 6 | propinv | dirichlet (1,1,1,1) |
| *Nad2*: alignment pos 3, codon pos 3 | | 336 | 6 | propinv | dirichlet (1,1,1,1) |
| *Co1*: alignment pos 1, codon pos 1 | | 173 | 6 | gamma | fixed (equal) |
| *Co1*: alignment pos 2, codon pos 2 | | 172 | 1 | equal | dirichlet (1,1,1,1) |
| *Co1*: alignment pos 3, codon pos 3 | | 172 | 6 | gamma | dirichlet (1,1,1,1) |
| *tRNAs*: *His, Ile, Met, Trp, Ala, Asn, Tyr* | | 421 | 6 | gamma | dirichlet (1,1,1,1) |
| *Nad4*: alignment pos 1, codon pos 1 | | 210 | 6 | gamma | dirichlet (1,1,1,1) |
| *Nad4*: alignment pos 2, codon pos 2 | | 210 | 2 | propinv | dirichlet (1,1,1,1) |
| *Nad4*: alignment pos 3, codon pos 3 | | 209 | 2 | propinv | dirichlet (1,1,1,1) |
| *Cytb*: alignment pos 1, codon pos 2 | | 217 | 1 | equal | dirichlet (1,1,1,1) |
| *Cytb*: alignment pos 2, codon pos 3 | | 217 | 2 | equal | dirichlet (1,1,1,1) |
| *Cytb*: alignment pos 3, codon pos 1 | | 217 | 1 | equal | dirichlet (1,1,1,1) |
