# Supplementary material for: Evolution of Gigantism in Amphiumid Salamanders
Source: PLoS One. 2009 May 20;4(5):e5615. doi: 10.1371/journal.pone.0005615 (PMC2680017; doi:10.1371/journal.pone.0005615)
Supplement: Table S5 — Species and Genbank numbers for Bayesian phylogenetic analysis of Rag1 that was used for divergence time estimates in r8s. (0.04 MB DOC) [file pone.0005615.s005.doc]

| **Family** | **Species** | **Genbank**  **Accession *Rag1*** |
| --- | --- | --- |
| Ichthyophuidae (Caecilian) | *Ichthyophis* sp*.* | AY650147 |
| Ascaphidae (Anuran) | *Ascaphus montanus* | AY650146 |
| Cryptobranchidae | Andrias davidianus | AY650142 |
| Salamandridae | *Salamandra salamandra* | AY650135 |
| Salamandridae | *Taricha rivularis* | AY650133 |
| Ambystomatidae | *Ambystoma mexicanum* | EF551561 |
| Dicamptodontidae | *Dicamptodon tenebrosus* | AY650132 |
| Rhyacotritonidae | *Rhyacotriton variagatus* | AY691693 |
| Amphiumidae | *Amphiuma means* | AY650127 |
| Amphiumidae | *Amphiuma pholeter* | AY650128 |
| Amphiumidae | *Amphiuma tridactylum* | FJ951369 |
| Plethodontidae | *Aneides aeneus* | AY691701 |
| Plethodontidae | *Aneides lugubris* | AY650118 |
| Plethodontidae | *Bolitoglossa helmrichi* | AY650124 |
| Plethodontidae | *Desmognathus brimleyorum* | AY691697 |
| Plethodontidae | *Ensatina eschscholtzii* | AY691743 |
| Plethodontidae | *Eurycea bislineata* | AY691706 |
| Plethodontidae | *Hemidactylium scutatum* | AY691711 |
| Plethodontidae | *Phaeognathus hubrichti* | AY691700 |
| Plethodontidae | *Plethodon cinereus* | AY691703 |

*All tree partitions were set to: nst=6, rates=invgamma, statefreqpr=dirichlet (1,1,1,1).
