# Supplementary material for: Evolution of Gigantism in Amphiumid Salamanders
Source: PLoS One. 2009 May 20;4(5):e5615. doi: 10.1371/journal.pone.0005615 (PMC2680017; doi:10.1371/journal.pone.0005615)
Supplement: Table S6 — External calibration points used for nonparametric rate smoothing analysis of Rag1 using r8s. Points are plotted on Figures S1 and S2. (0.04 MB DOC) [file pone.0005615.s006.doc]

| #- Fossil and/or description of calibration point | **Date** | **Reference** |
| --- | --- | --- |
| **1*** - *Chunerpeton tianyiensis*, fixed MRCA of cryptobranchids and salamandroids | 161 MYA | [46] |
| **2 ***- Estimated molecular divergence of cryptobranchids and salamandroids. | 250 MYA | [30, 31, 47] |
| **3** - *Proamphiuma cretacea*, minimum MRCA of amphiumids and plethodontids | 65.5 MYA | [48, 49] |
| **4** - *Dicamptodon antiquuis*, minimum MRCA of dicamptodontids and ambystomatids | 58 MYA | [50] |
| **5**- *Aneides* sp., minimum MRCA of *Aneides* and desmognathines | 19 MYA | [51] |
| **6** - *Aneides lugubris*, minimum MRCA *Aneides* from eastern and western North America | 5 MYA | [52] |

MRCA = Most recent common ancestor

MYA = Million years ago

*Note: analyses are based on either fixing the root node (MRCA of cryptobranchids and salamandroids) at either 161 MYA (1) or 250 MYA (2). See methods section.
