# Supplementary figures and images for: Evolution of Gigantism in Amphiumid Salamanders
Source: PLoS One. 2009 May 20;4(5):e5615. doi: 10.1371/journal.pone.0005615 (PMC2680017; doi:10.1371/journal.pone.0005615)

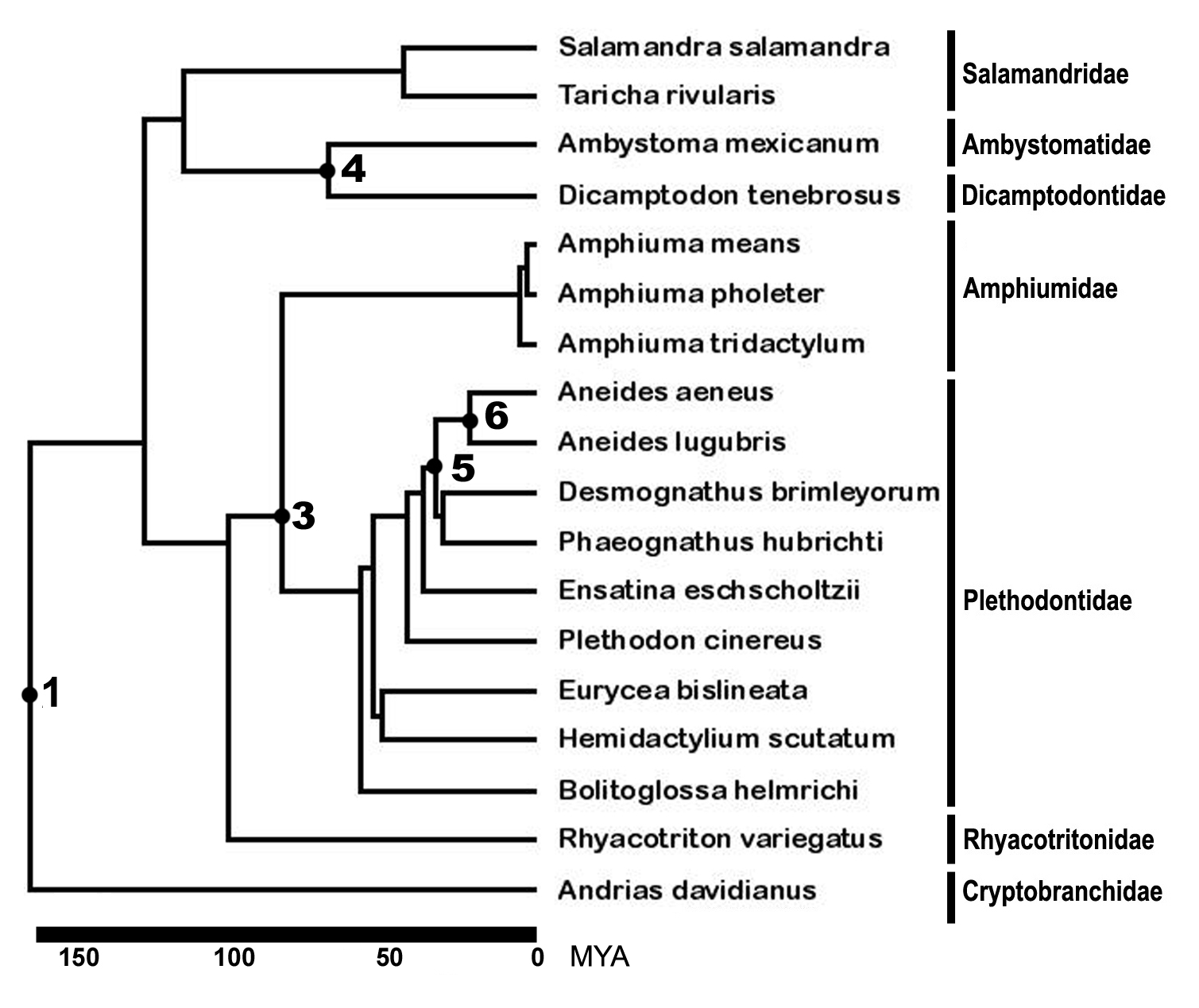

Supplement: Figure S1 — Chronogram of salamander families calculated in r8s based on Bayesian analysis of Rag1, fixing the basal node at 161 MYA, and four external calibration points (Table S5, S6). (5.76 MB TIF) [file pone.0005615.s007.tif]

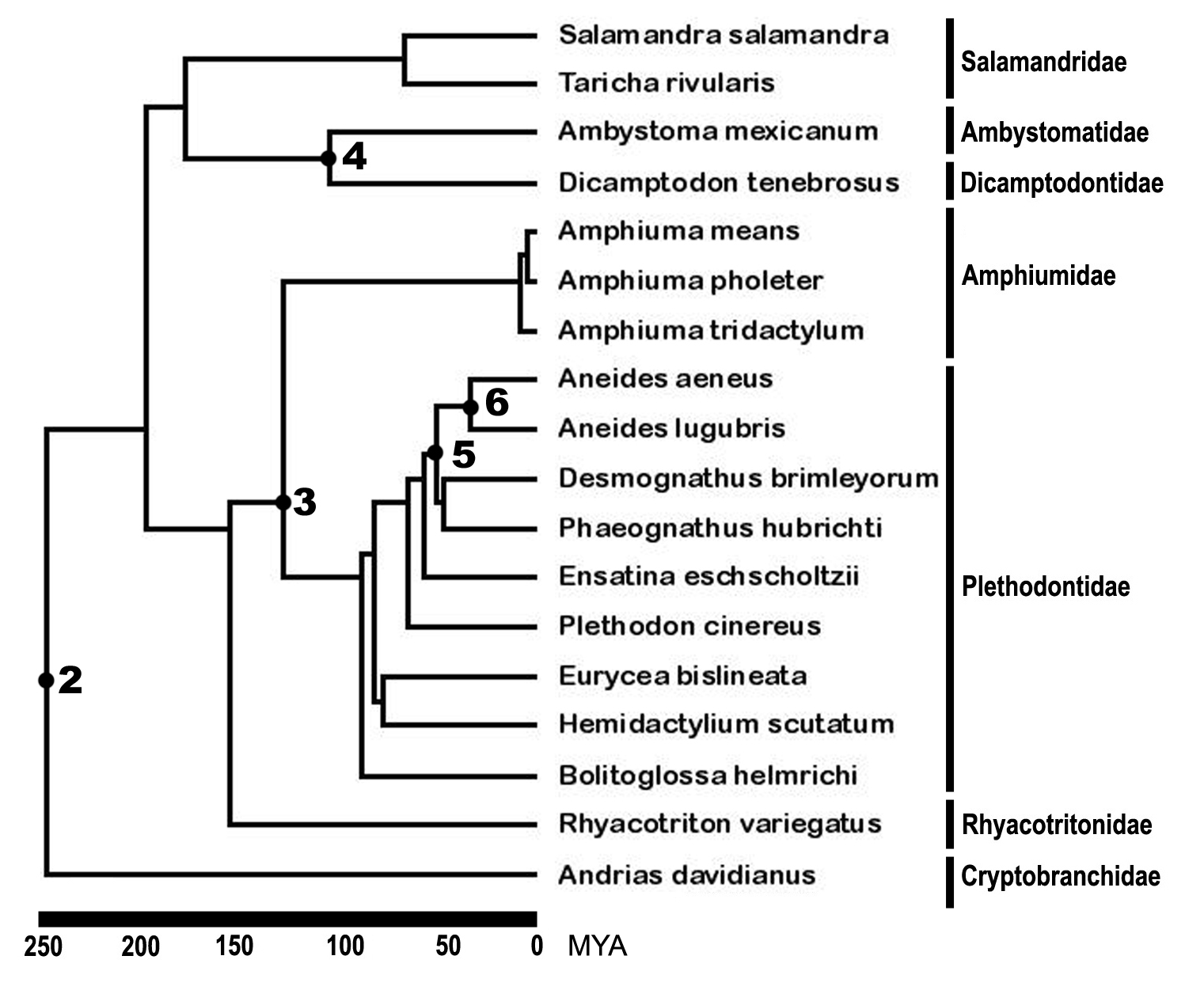

Supplement: Figure S2 — Chronogram of salamander families calculated in r8s based on Bayesian analysis of Rag1, fixing the basal node at 250 MYA, and four external calibration points (Table S5, S6). (5.73 MB TIF) [file pone.0005615.s008.tif]
